# Supplementary material for: Controlling reaction pathways of selective C–O bond cleavage of glycerol
Source: Nat Commun. 2018 Nov 5;9:4612. doi: 10.1038/s41467-018-07047-7 (PMC6218480; doi:10.1038/s41467-018-07047-7)
Supplement: Supplementary file 2 — Supplementary Information [file 41467_2018_7047_MOESM2_ESM.pdf]

Supplementary Information

Controlling reaction pathways of selective C-O bond cleavage of glycerol

Wan et al.

Supplementary Figures

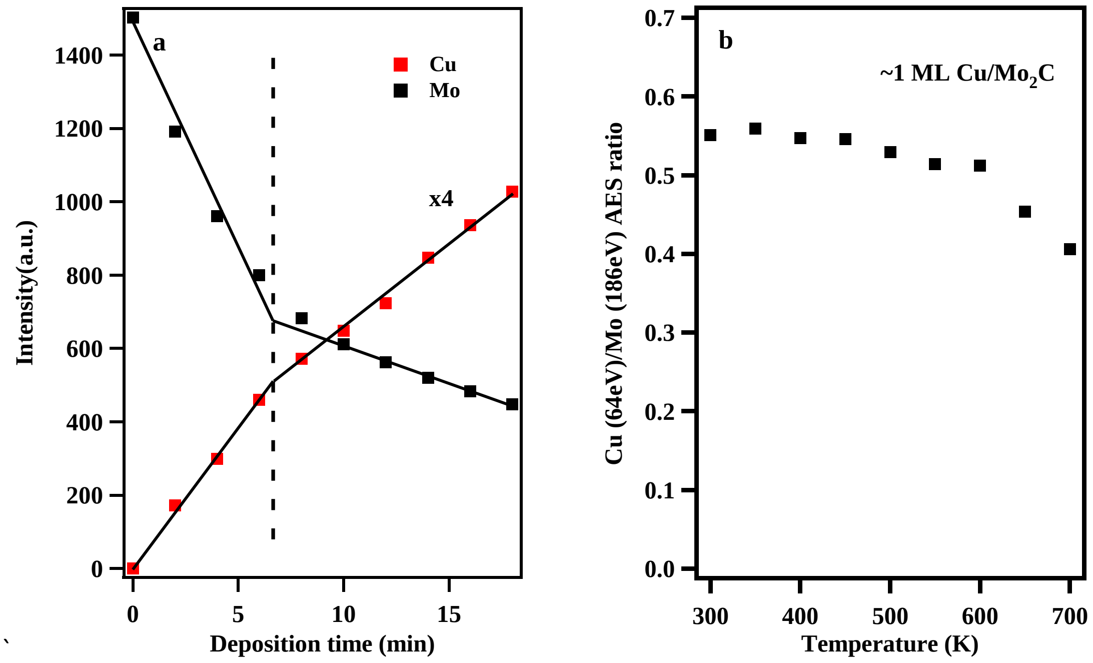

Supplementary Figure 1. AES measurements of Cu(64 eV) and Mo(186 eV) for the Cu/Mo_2C/Mo(110) surfaces. **a** Cu deposition on Mo_2C/Mo(110) at 300 K as a function of deposition time, **b** Cu(64 eV)/Mo(186 eV) ratio for 1 ML Cu/ Mo_2C/Mo(110) as a function of annealing temperature

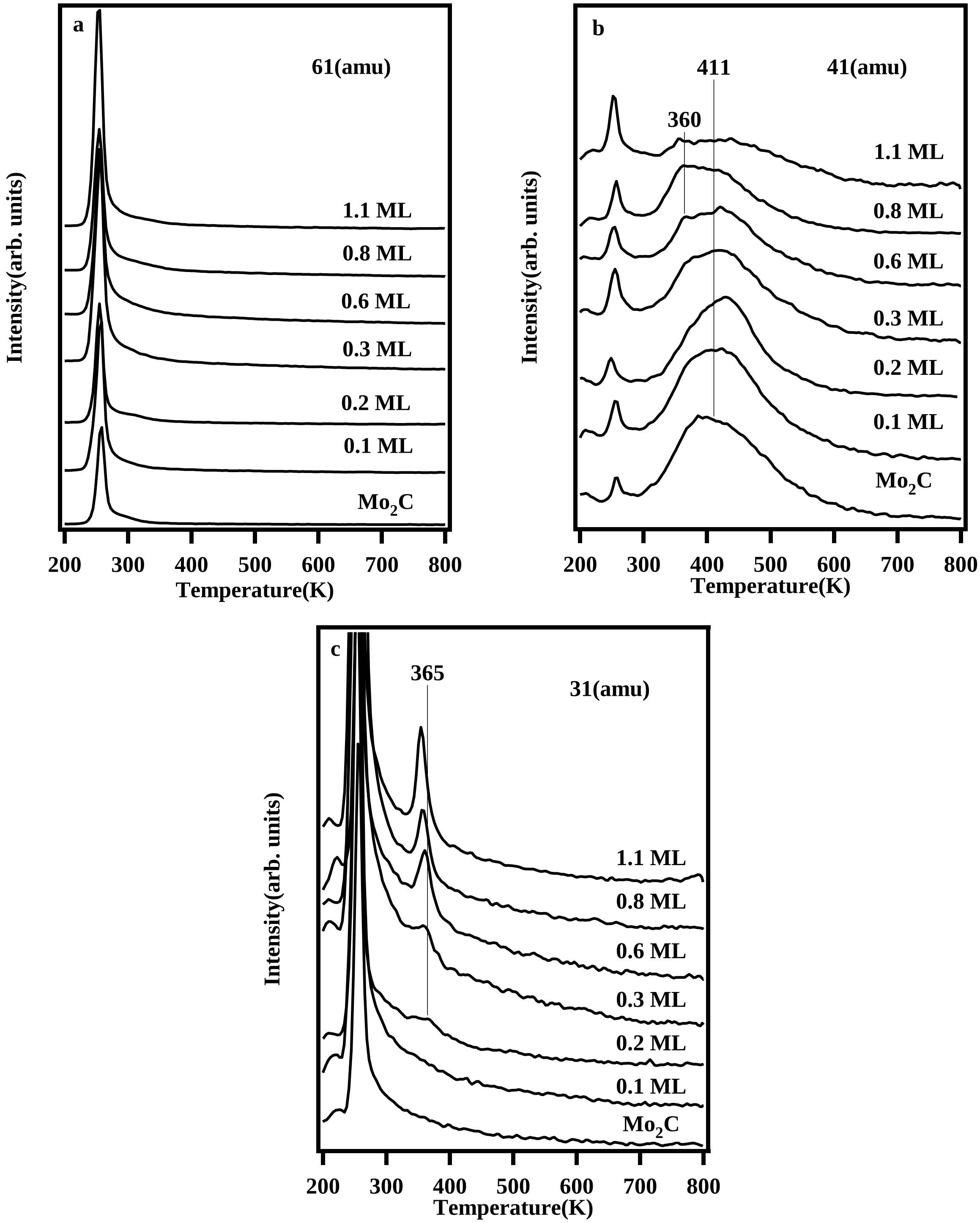

Supplementary Figure 2. Gas-phase products of the TPD experiments. 4 L glycerol was exposed on hydrogen pre-dosed Cu/Mo_2C surfaces with different Cu coverages. Spectra of **a** glycerol (m/z=61), **b** propylene (m/z=41), and **c** acetol (m/z=31)

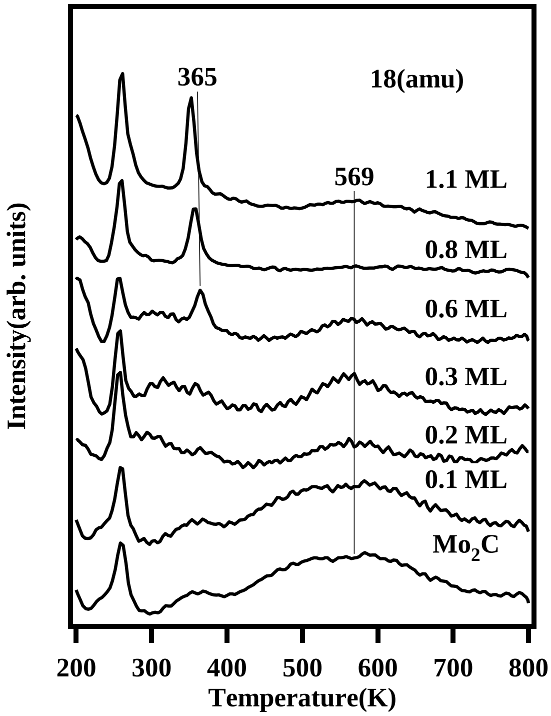

Supplementary Figure 3. TPD spectra of water (m/z=18) with an exposure of 4 L glycerol on hydrogen pre-dosed Cu/Mo_2C surfaces with different Cu coverages

In the Supplementary Figure 3, the sharp peak at 260 K was from the cracking pattern of glycerol. A sharp peak at 365 K was observed on the Cu-terminated surface, and a broad peak at 569 K was observed on the Mo_2C surface.

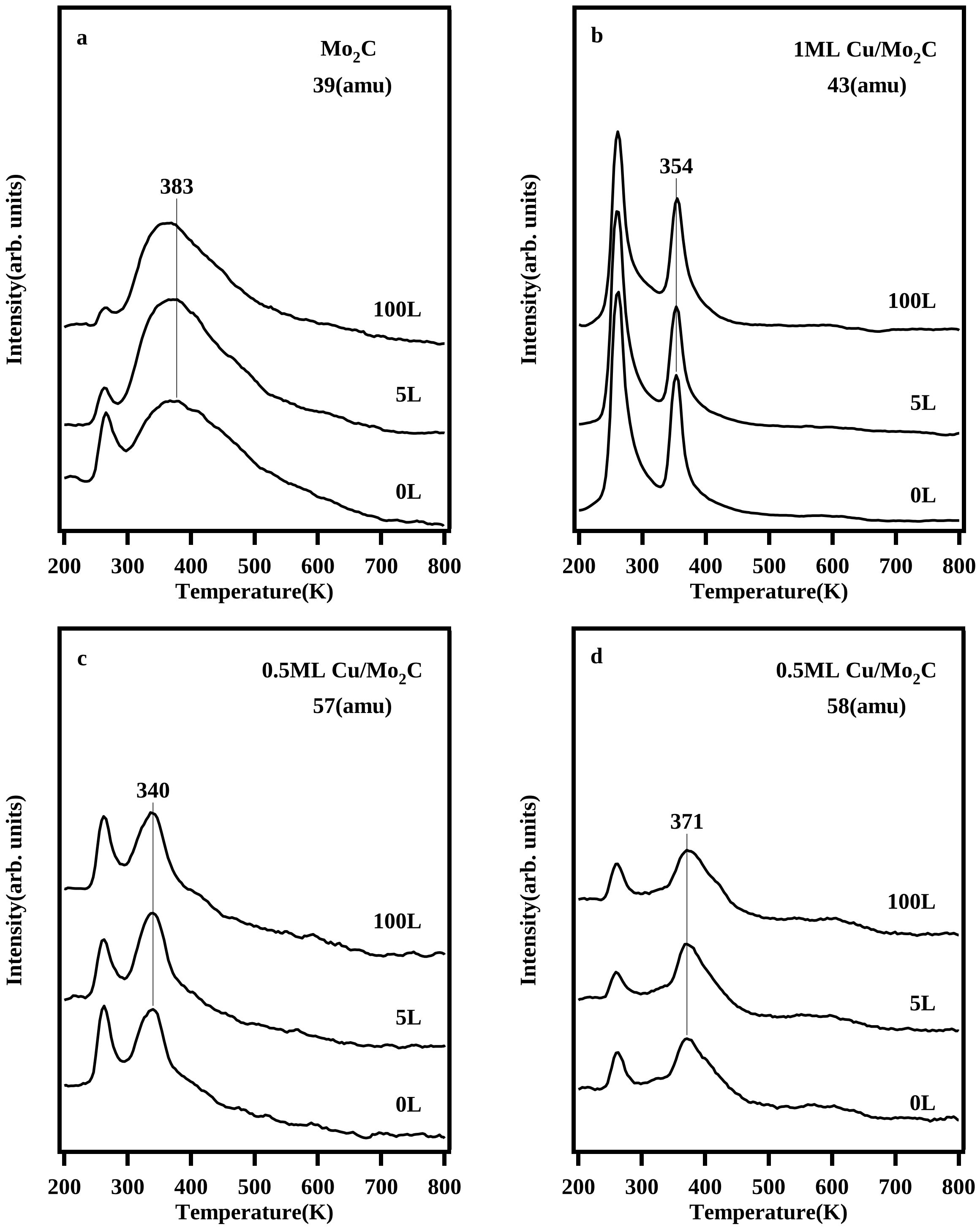

Supplementary Figure 4. TPD results of glycerol on Cu/Mo_2C surfaces with 0 L, 5 L and 100 L H_2 pre-dosed. Spectra of **a** propylene desorption from the Mo_2C surface, **b** acetol desorption from the 1 ML Cu/Mo_2C surface, **c** allyl-alcohol, and **d** propanal desorption from the 0.5 ML Cu/Mo_2C surface

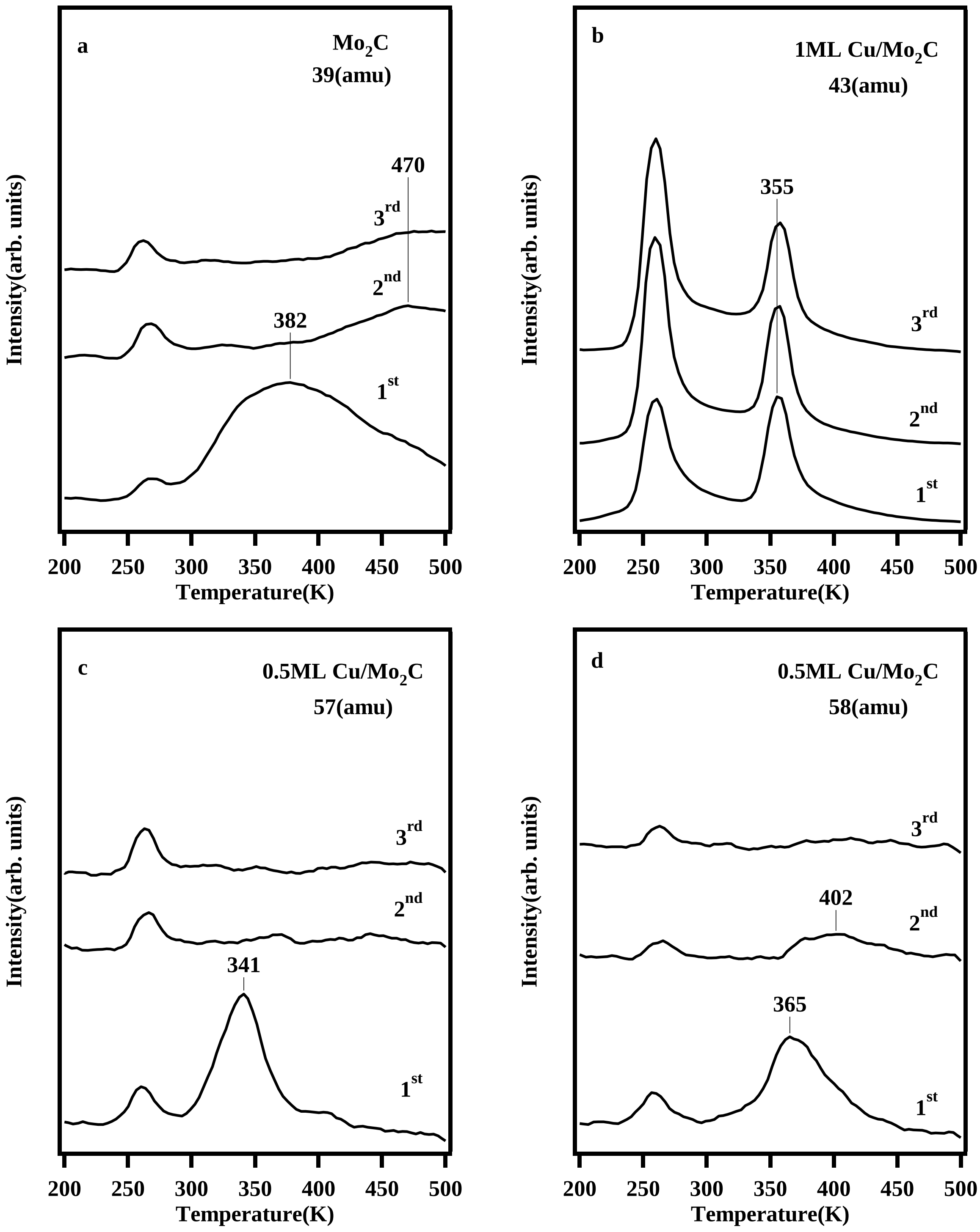

Supplementary Figure 5. TPD results of sequential experiments. Spectra of **a** propylene desorption from the Mo_2C surface, **b** acetol desorption from the 1 ML Cu/Mo_2C surface, **c** allyl-alcohol, and **d** propanal desorption from the 0.5 ML Cu/Mo_2C surface

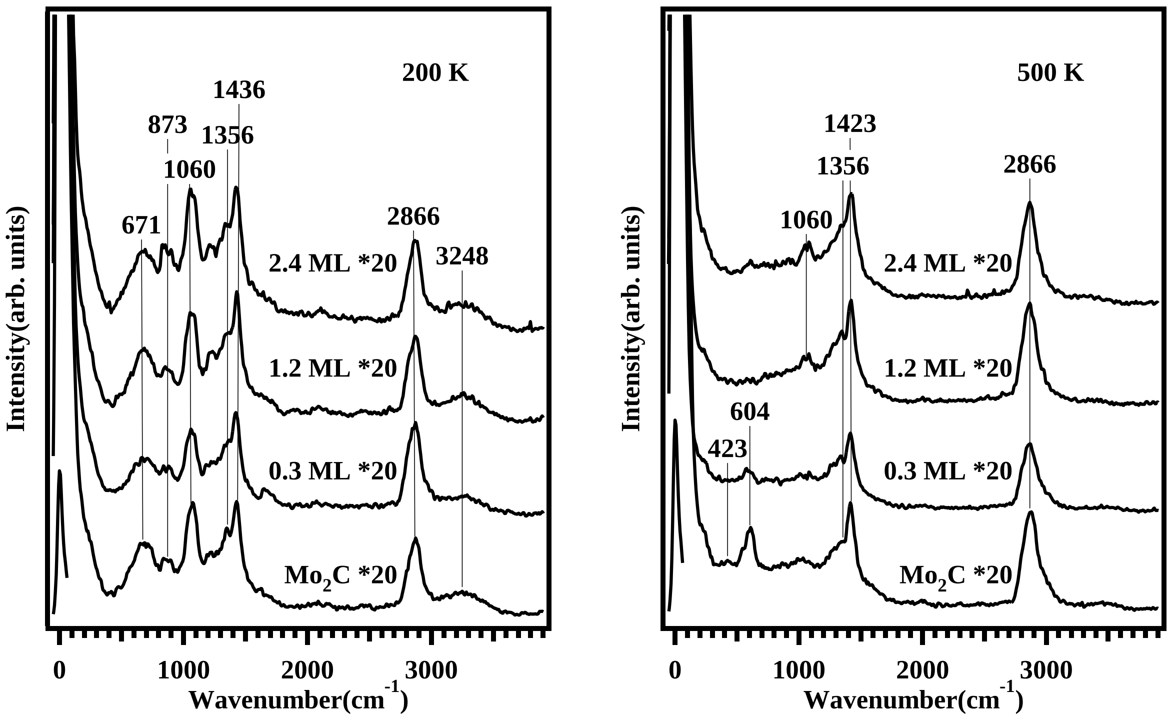

Supplementary Figure 6. HREELS measurements of the surface intermediates. Glycerol was exposed on H_2 pre-dosed Mo_2C/Mo(110), 0.3 ML Cu/Mo_2C/Mo(110), 1.2 ML Cu/Mo_2C/Mo(110), and 2.4 ML Cu/Mo_2C/Mo(110). Spectra at **a** 200 K and **b** 500 K.

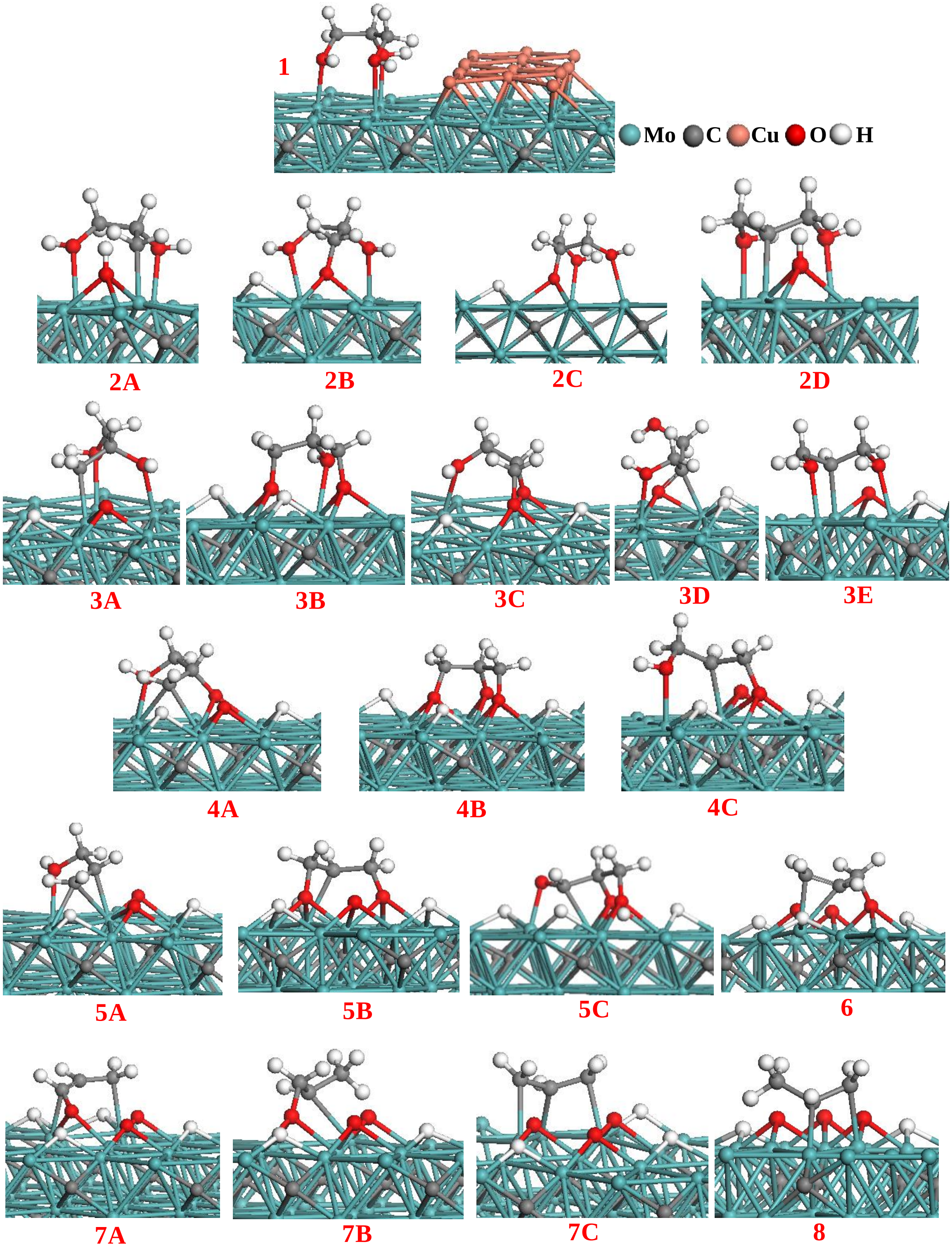

Supplementary Figure 7. Side view of intermediate structures involved in the glycerol deoxygenation pathways on the Mo sites of the $Cu/Mo_2C(0001)$ catalyst model. The numbers correspond to the states provided in Figure 4 of the paper. Cu atoms in the intermediates are omitted for clarity. Mo: blue; C: gray; O: red; H: white

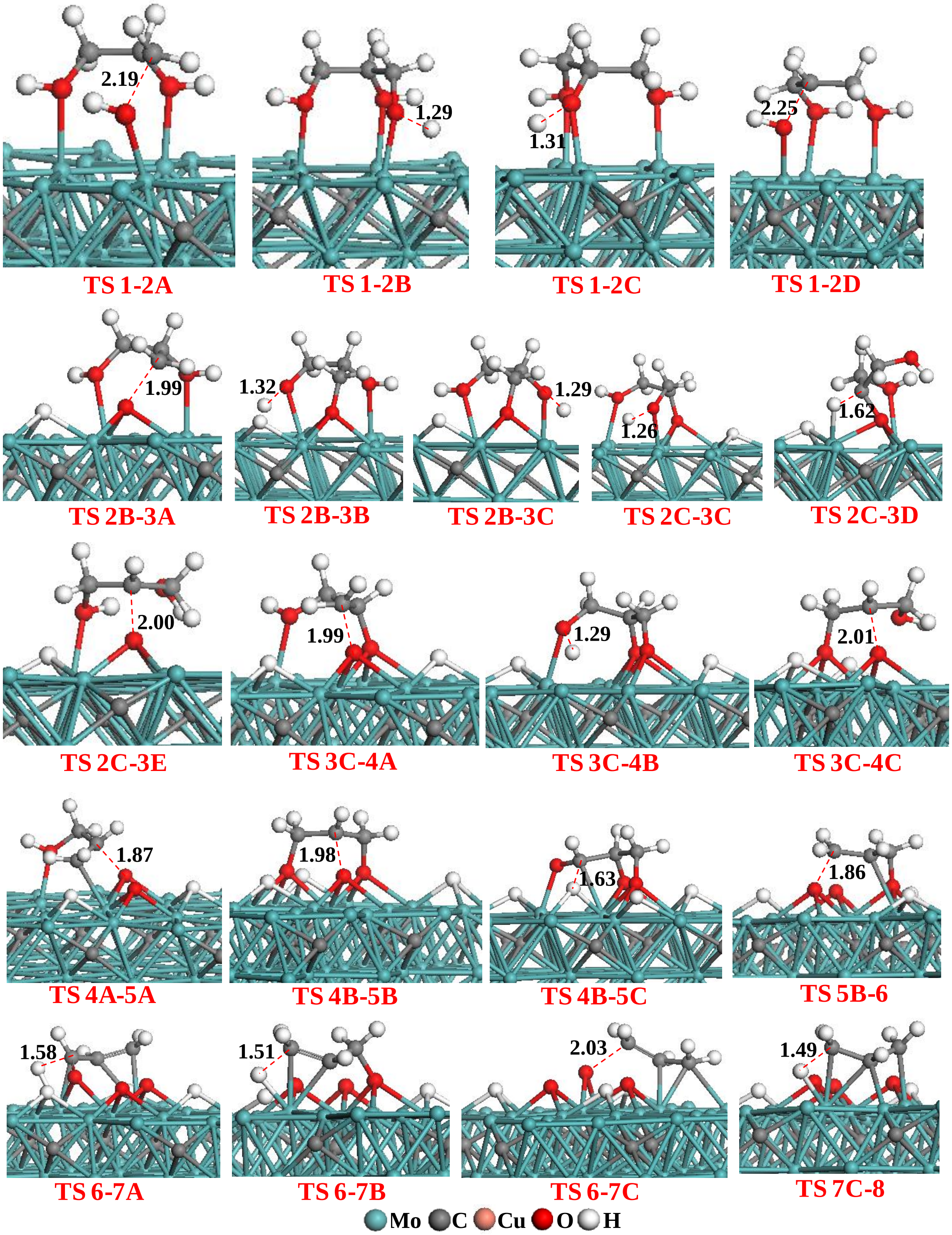

Supplementary Figure 8. Side view of the transition state (TS) structures involved in the glycerol deoxygenation pathways on the Mo sites of the Cu/Mo_2C(0001) catalyst model. The TS numbers correspond to the states provided in Figure 4 of the paper. Cu atoms are omitted for clarity. Bond distances shown next to the breaking/forming bonds are in Å. Mo: blue; C: gray; O: red; H: white

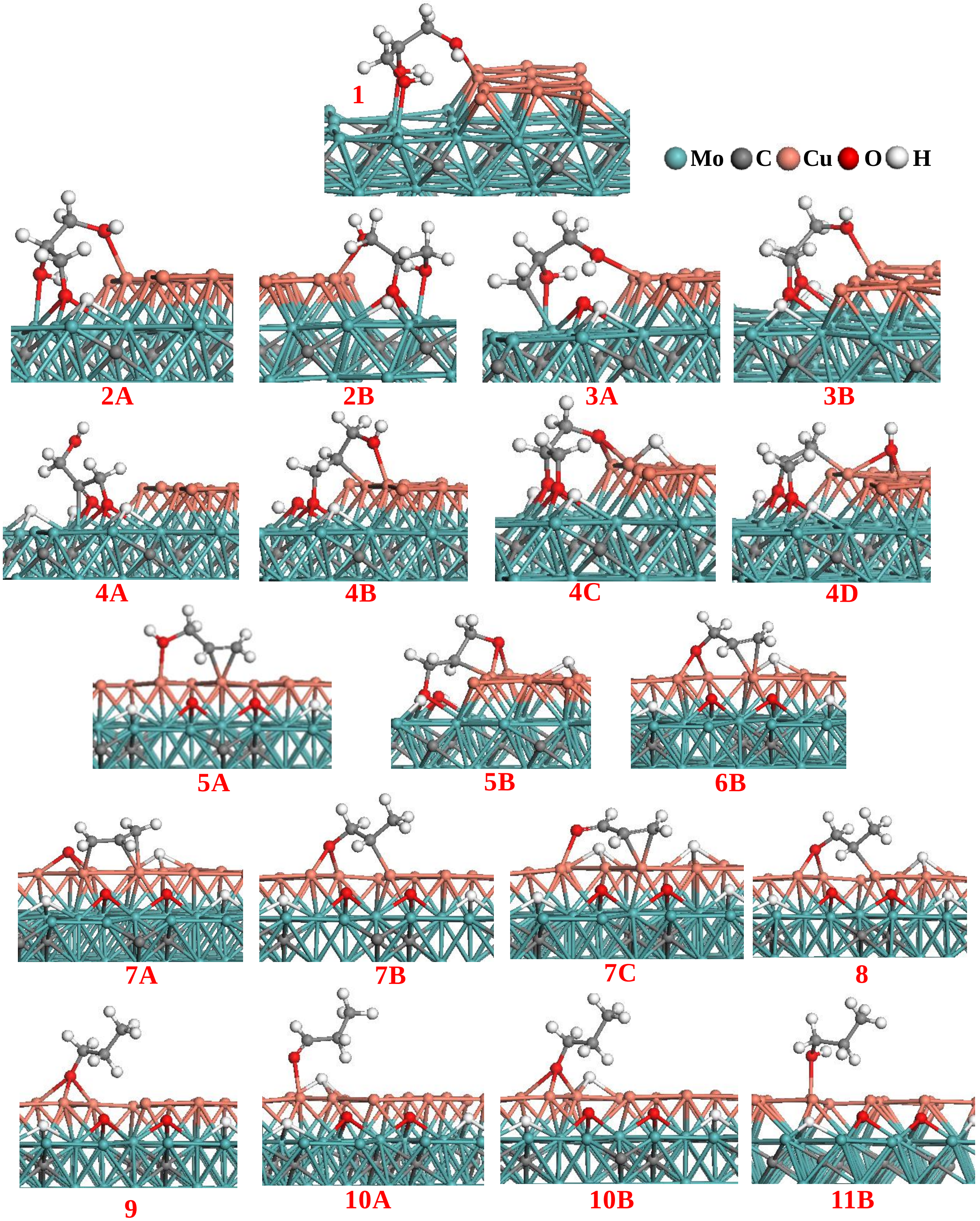

Supplementary Figure 9. Side view of intermediate structures involved in the glycerol deoxygenation pathways at the interface sites of the Cu/Mo_2C(0001) catalyst model. The numbers correspond to the states provided in Figure 5 of the paper. Mo: blue; C: gray; Cu: pink; O: red; H: white

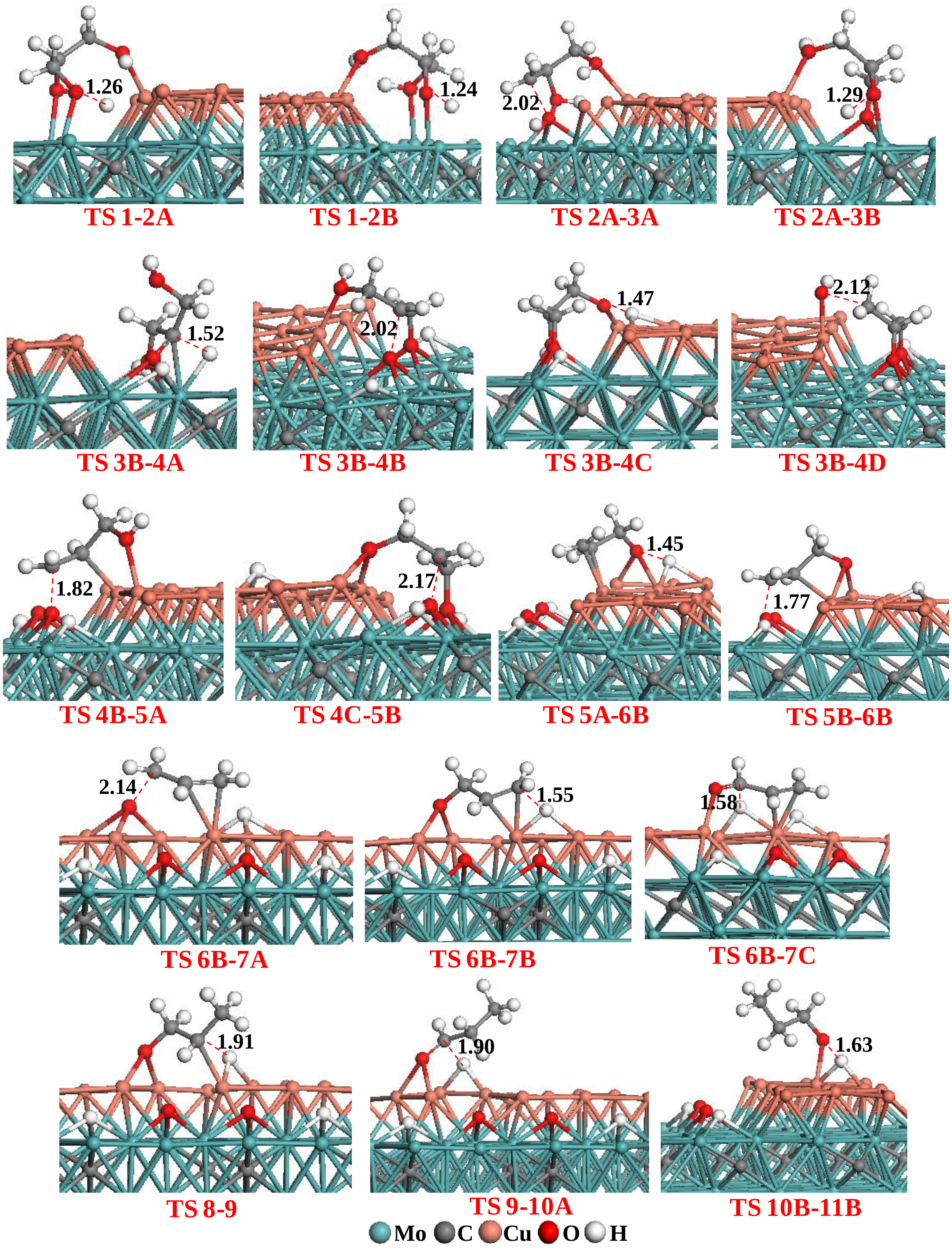

Supplementary Figure 10. Side view of the transition state (TS) structures involved in the glycerol deoxygenation pathways at the interface sites of the Cu/$Mo_2C(0001)$ catalyst model. The TS numbers correspond to the states provided in Figure 5 of the paper. Bond distances shown next to the breaking/forming bonds are in Å. Mo: blue; C: gray; Cu: pink; O: red; H: white

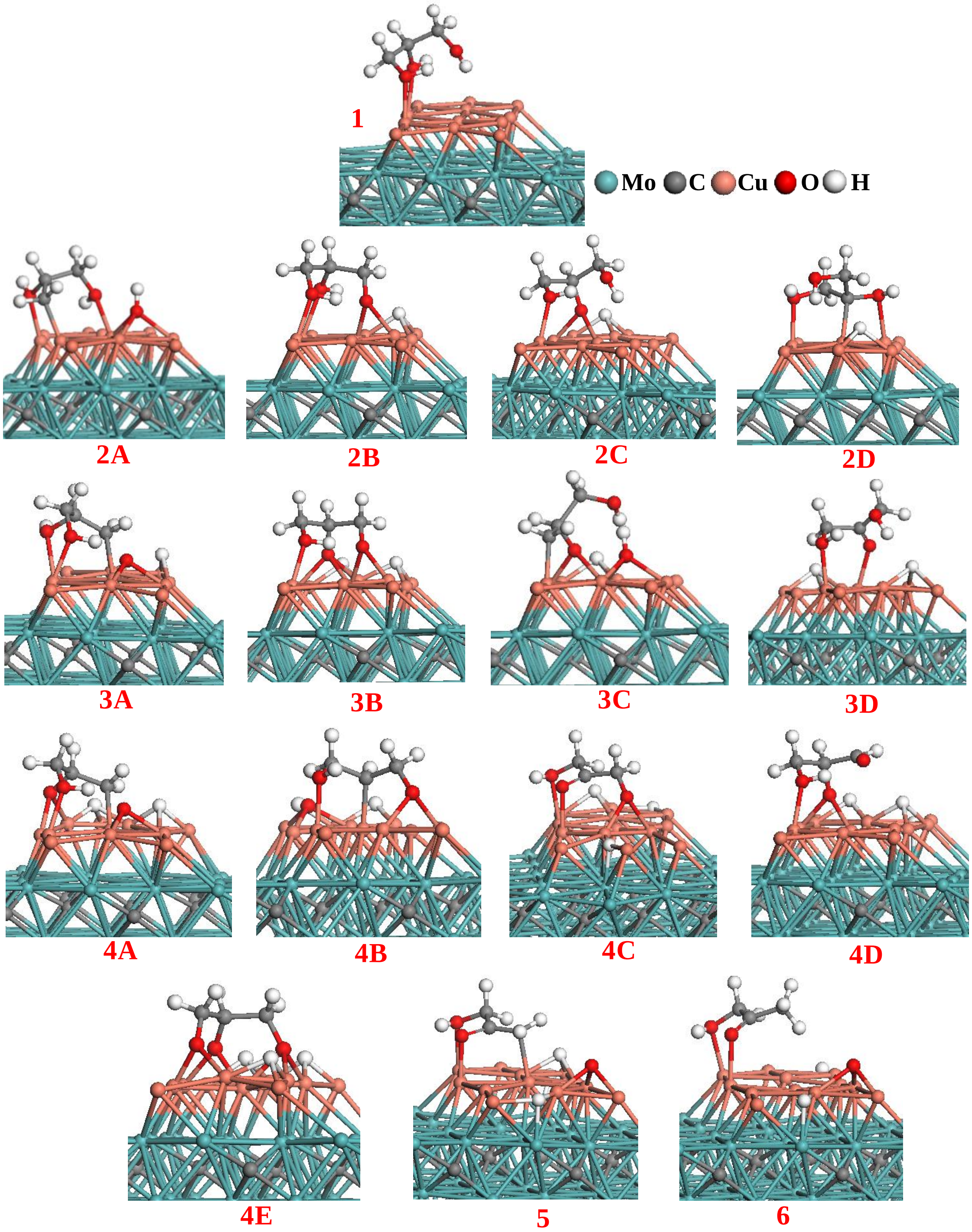

Supplementary Figure 11. Side view of intermediate structures involved in the glycerol deoxygenation pathways on the Cu sites of the Cu/Mo_2C(0001) catalyst model. The numbers correspond to the states provided in Figure 6 of the paper. Mo: blue; C: gray; Cu: pink; O: red; H: white

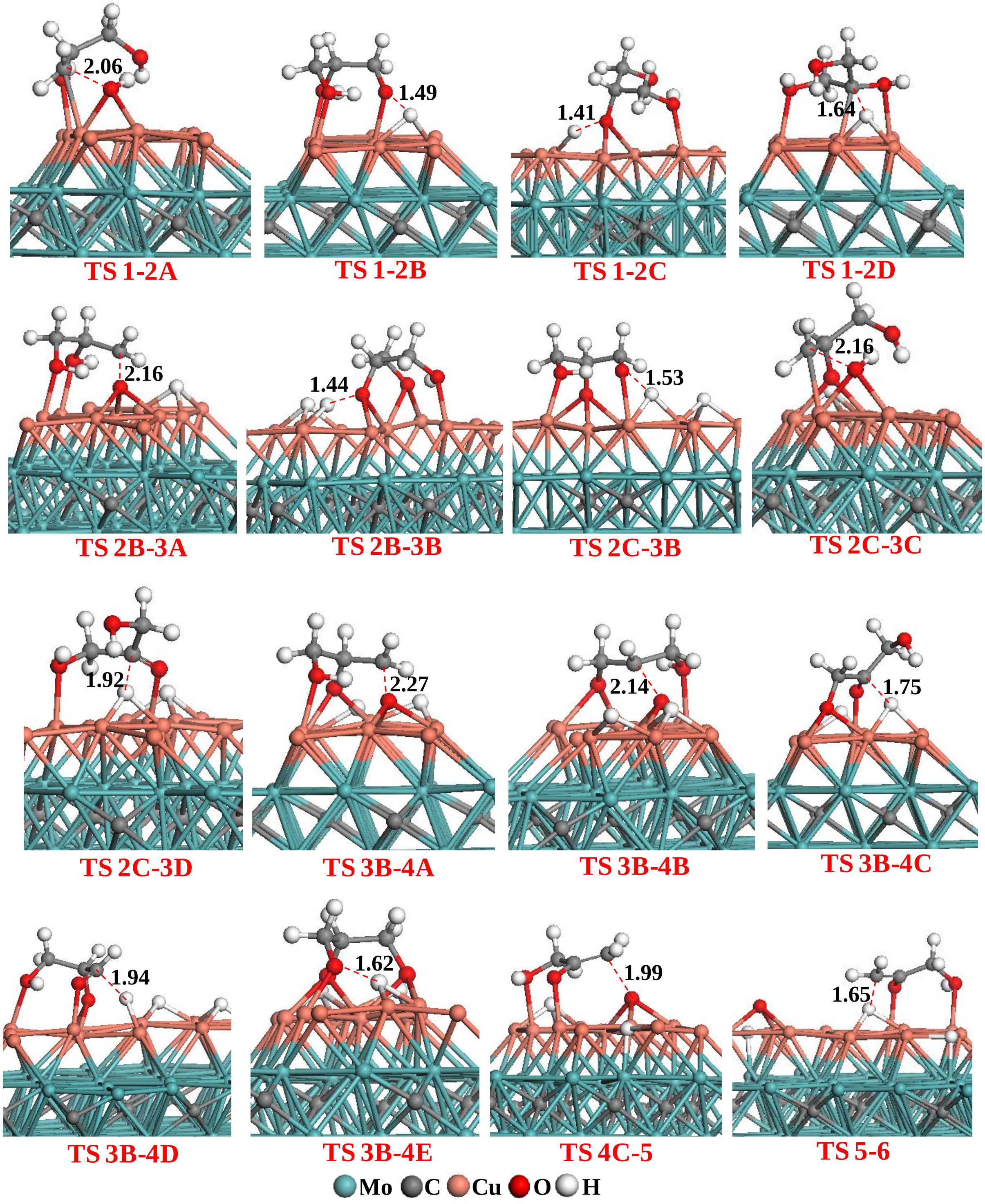

Supplementary Figure 12. Side view of the transition state (TS) structures involved in the glycerol deoxygenation pathways on the Cu sites of the Cu/Mo_2C(0001) catalyst model. The TS numbers correspond to the states provided in Figure 6 of the paper. Bond distances shown next to the breaking/forming bonds are in Å. Mo: blue; C: gray; Cu: pink; O: red; H: white

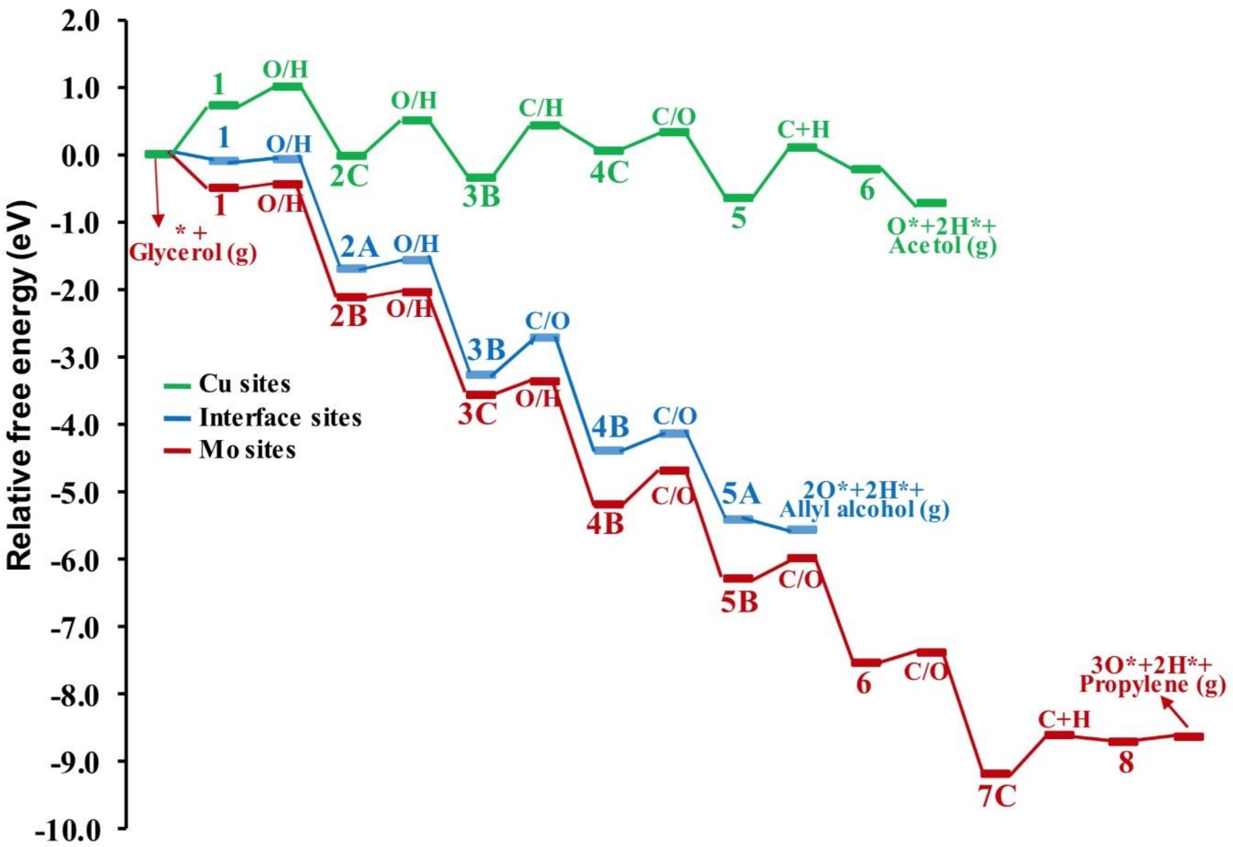

Supplementary Figure 13. Free energy profiles for the minimum energy pathways of glycerol decomposition on different active sites of the $Cu/Mo_2C(0001)$ catalyst model (T = 350 K; P_{gas} = 10^{-9} atm). All energies are with reference to the sum of the energies of gas phase glycerol and the initial catalyst model. The numbering of intermediate states corresponds to the states in Figures 4, 5, and 6 of the manuscript. The respective bond dissociations/associations are shown at the transition states

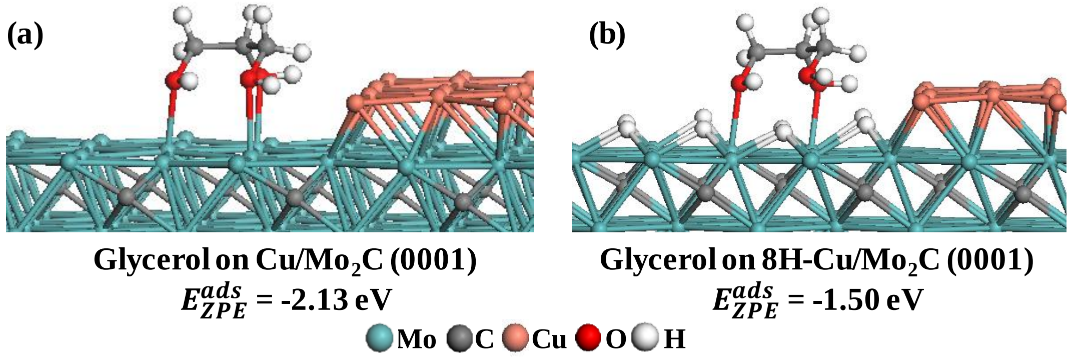

Supplementary Figure 14. Side view of glycerol adsorbed structures on the Mo sites of the $Cu/Mo_2C(0001)$ catalyst model. The zero-point corrected adsorption energies of glycerol are given below the structures. **a** adsorption structures in the absence of H atoms and **b** adsorption structures in the presence of H atoms. Mo: blue; C: gray; Cu: pink; O: red; H: white

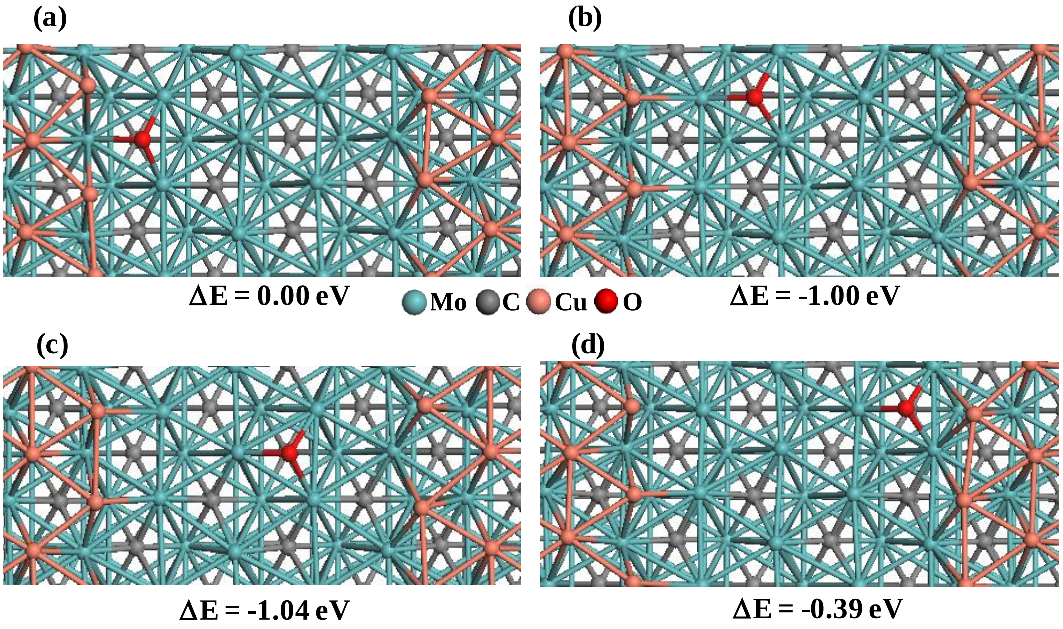

Supplementary Figure 15. Top view of the Cu/Mo_2C(0001) catalyst model with oxygen atoms adsorbed on similar C-vacant 3-fold hollow sites. The energies shown under each structure are the relative energies with respect to structure (a). The unit cell is expanded to show a clear view of the position of the adsorbed oxygen atoms. Mo: blue; C: gray; Cu: pink; O: red

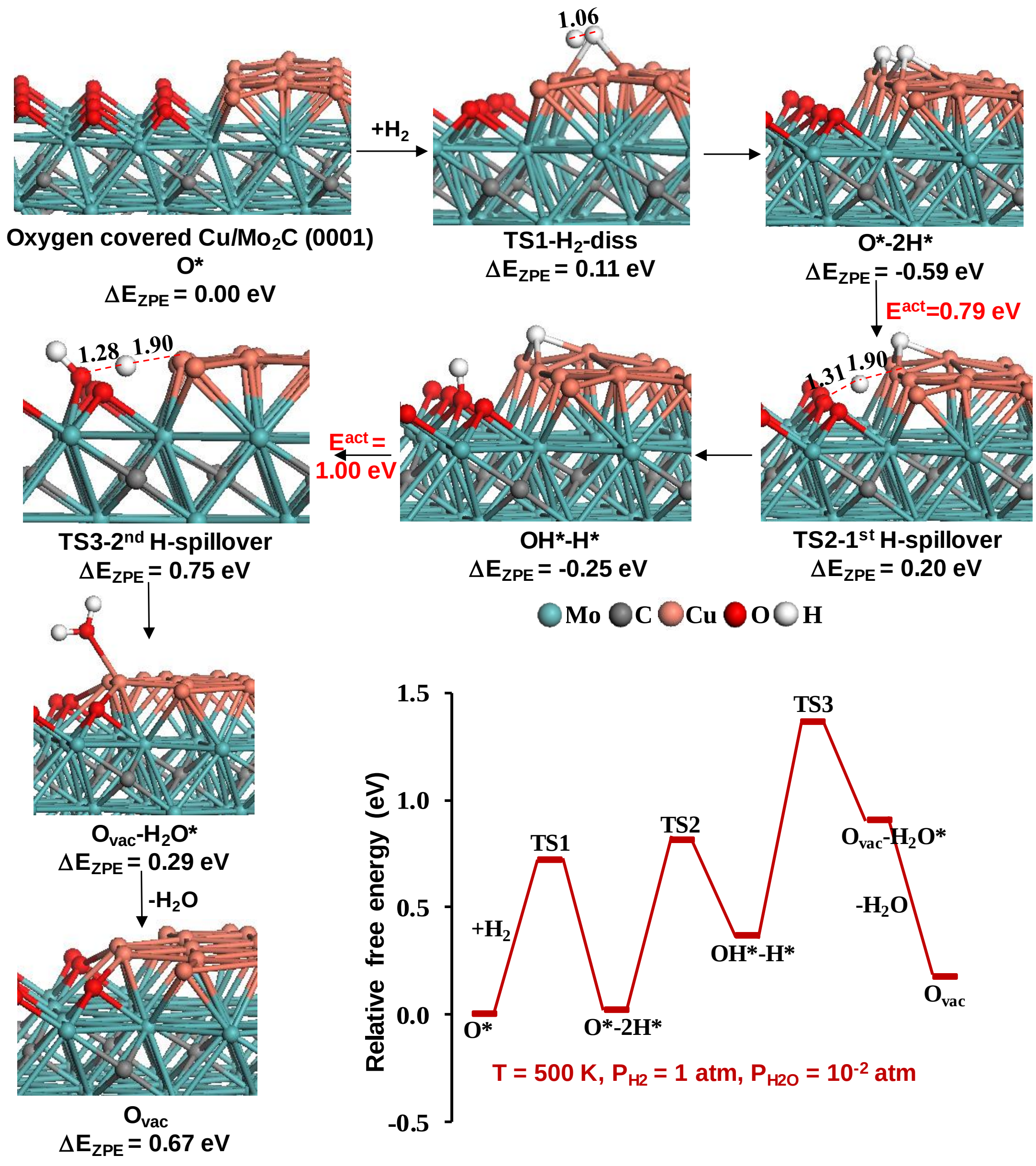

Supplementary Figure 16. Reaction pathways calculated for the removal of interface oxygen from the oxygen covered Cu/Mo_2C(0001) catalyst model. Bond distances shown next to the breaking/forming bonds are in Å. Mo: blue; C: gray; Cu: pink; O: red

Supplementary Tables

Supplementary Table 1. Zero-point energy corrected reaction energies (ΔE_{ZPE}) and activation barriers (E_{ZPE}^{act}) calculated for the formation of H_2O on different active sites of the Cu/Mo_2C (0001) catalyst model

Reaction	ΔE_{ZPE} (eV)	E_{ZPE}^{act} (eV)
**Mo sites of the Cu/Mo_2C catalyst**		
(1) O* + H* → OH* + *	1.20	2.08
(2) OH* + H* → H_2O* + *	1.52	1.90
(3) O* + H_2(g) → OH* + H*	-0.56	0.38
(4) OH* + OH* → H_2O* + O*	0.42	0.75
(5) H_2O* → H_2O(g) + *	0.94	0.94
**Cu/Mo_2C interface sites**		
(6) H_2(g) + 2$*_{Cu}$ → 2H_{Cu}*	-0.51	0.17
(7) 2H_{Cu}* + $O*_{Mo}$ → OH_{Mo}*+ H_{Cu}* + $*_{Cu}$	0.47	0.92
(8) OH_{Mo}*+ H_{Cu}* → H_2O_{Mo}* + $*_{Cu}$	1.03	1.12
(9) O_{Mo}*+ H_2(g) → OH_{Mo}*+ H_{Mo}*	-0.67	0.39
(10) OH_{Mo}*+ OH_{Mo}* → H_2O_{Mo}* + O_{Mo}*	0.44	0.61
(11) H_2O_{Mo}* → H_2O(g) + $*_{Mo}$	0.82	0.82
**Cu sites of the Cu/Mo_2C catalyst**		
(12) O* + H* → OH* + *	-0.48	1.05
(13) OH* + H* → H_2O* + *	0.79	1.36
(14) O* + H_2(g) → OH* + H*	-0.81	0.33
(15) OH* + OH* → H_2O* + O*	0.42	0.44
(16) H_2O* → H_2O(g) + *	0.35	0.35

Supplementary Table 2. Yields for the three reactions of glycerol from Scheme 1 from TPD experiments (molecules per metal atom)

	Mo_2C	0.1 ML	0.2 ML	0.3 ML	0.6 ML	0.8 ML	1.1 ML
Reaction 1	0.068	0.063	0.057	0.049	0.031	0.030	0.013
Reaction 2	0.008	0.017	0.026	0.032	0.041	0.020	0.008
Reaction 3	0.000	0.000	0.014	0.018	0.045	0.046	0.062
Total	0.075	0.081	0.096	0.099	0.117	0.096	0.083

Supplementary Table 3. Vibrational mode assignment for glycerol molecule

**Mode**	**Frequency (cm^{-1})**		
	**Ethylene glycol on Mo_2C[1]**	**Mo_2C/Mo(110)[a]**	**Cu/Mo_2C/Mo(110)[a]**
$\boldsymbol{\delta(MO)}$	--	430	--
$\boldsymbol{\nu(MO)}$	--	570	--
$\boldsymbol{\tau}$**(OH)**	744	671	671
$\boldsymbol{\nu}$**(CC)**	879	859	852
$\boldsymbol{\nu}$**(CO)**	1069	1060	1060
$\boldsymbol{\rho_w}$**(CH_2)**	1360	1356	1342
$\boldsymbol{\delta}$**(CH_2)**	1441	1430	1430
$\boldsymbol{\nu_{as}}$**(CH)**	2916	2873	2873
$\boldsymbol{\nu}$**(OH)**	3085	3248	3248

Abbreviation: δ – deformation, ν – stretching, τ – twisting, ρ_t – rocking, ρ_w - wagging, as-asymmetric

a. This work

The HREELS spectrum of ethylene glycol on Mo_2C was used as a reference to assign the vibrational modes of glycerol on Mo_2C/Mo(110) and Cu/Mo_2C/Mo(110) since glycerol and ethylene glycol have similar structures.

Supplementary Table 4. Zero-point energy corrected reaction energies (ΔE_{ZPE}**)** and forward activation barriers (E_{ZPE}^{act}) for all elementary steps considered in the glycerol decomposition mechanism on Mo sites of the Cu/Mo_2C (0001) catalyst model

**Reaction**[a]	$\boldsymbol{\Delta E_{ZPE}}$ **(eV)**	$\boldsymbol{E_{ZPE}^{act}}$ **(eV)**
CH_2OH-CHOH-CH_2OH (g) + * → CH_2OH-CHOH-CH_2OH* (1)	-2.13	0.00
CH_2OH-CHOH-CH_2OH* (1) → CH_2OH-CHOH-CH_2* + OH* (2A)	-1.54	1.11
CH_2OH-CHOH-CH_2OH* (1) → CH_2OH-CHOH-CH_2O* + H* (2B)	-1.65	0.03
CH_2OH-CHOH-CH_2OH* (1) → CH_2OH-CHO-CH_2OH* + H* (2C)	-1.70	0.28
CH_2OH-CHOH-CH_2OH* (1) → CH_2OH-CH-CH_2OH* + OH* (2D)	-1.56	0.98
CH_2OH-CHOH-CH_2O* + H* (2B) → CH_2OH-CHOH-CH_2* +H* +O* (3A)	-1.33	0.89
CH_2OH-CHOH-CH_2O* + H* (2B) → CH_2O-CHOH-CH_2O* + 2H* (3B)	-1.32	0.14
CH_2OH-CHOH-CH_2O* + H* (2B) → CH_2OH-CHO-CH_2O* + 2H* (3C)	-1.45	0.09
CH_2OH-CHO-CH_2OH* + H* (2C) → CH_2OH-CHO-CH_2O* + 2H* (3C)	-1.40	0.20
CH_2OH-CHO-CH_2OH* + H* (2C) → CH_2OH-CO-CH_2OH* + 2H* (3D)	0.19	1.30
CH_2OH-CHO-CH_2OH* + H* (2C) → CH_2OH-CH-CH_2OH* +H* +O* (3E)	-1.13	1.17
CH_2OH-CHO-CH_2O* + 2H* (3C) → CH_2OH-CHO-CH_2* +2H* +O* (4A)	-1.35	0.68
CH_2OH-CHO-CH_2O* + 2H* (3C) → CH_2O-CHO-CH_2O* + 3H* (4B)	-1.60	0.21
CH_2OH-CHO-CH_2O* + 2H* (3C) → CH_2OH-CH-CH_2O* +2H* +O* (4C)	-1.18	0.73
CH_2OH-CHO-CH_2* +2H* +O* (4A) → CH_2OH-CH=CH_2* +2H* +2O* (5A)	-1.05	0.24
CH_2O-CHO-CH_2O* + 3H* (4B) → CH_2O-CH-CH_2O* +3H* +O* (5B)	-1.13	0.48
CH_2O-CHO-CH_2O* + 3H* (4B) → CHO-CHO-CH_2O* +4H* (5C)	-0.40	0.75
CH_2O-CH-CH_2O* +3H* +O* (5B) → CH_2O-CH=CH_2* +3H* +2O* (6)	-1.24	0.31
CH_2O-CH=CH_2* +3H* +2O* (6) → CHO-CH=CH_2* +4H* +2O* (7A)	0.20	0.85
CH_2O-CH=CH_2* +3H* +2O* (6) → CH_2O-CH-CH_3* +2H* +2O* (7B)	0.43	0.42
CH_2O-CH=CH_2* +3H* +2O* (6) → CH_2-CH=CH_2* +3H* +3O* (7C)	-1.64	0.15
CH_2-CH=CH_2* +3H* +3O* (7C) → CH_3-CH=CH_2* +2H* +3O* (8)	0.48	0.56
CH_3-CH=CH_2* +2H* +3O* (8) → CH_3-CH=CH_2 (g) + (2H*+3O*)(9)	1.50	0.00

[a] Each reactant or product state in the elementary reactions constitute one discrete state although they are written as having multiple species for clarity

Supplementary Table 5. Zero-point energy corrected reaction energies (ΔE_{ZPE}) and forward activation barriers (E_{ZPE}^{act}) for all elementary steps considered in the glycerol decomposition mechanism at interface sites of the Cu/Mo_2C (0001) catalyst model

**Reaction**[a]	ΔE_{ZPE} (eV)	E_{ZPE}^{act} (eV)
CH_2OH-CHOH-CH_2OH (g) + * → CH_2OH-CHOH-CH_2OH* (1)	-1.76	0.00
CH_2OH-CHOH-CH_2OH* (1) → CH_2OH-CHOH-CH_2O* + H_{Mo}* (2A)	-1.58	0.04
CH_2OH-CHOH-CH_2OH* (1) → CH_2OH-CHO-CH_2OH* + H_{Mo}* (2B)	-1.62	0.20
CH_2OH-CHOH-CH_2O* +H_{Mo}* (2A) → CH_2OH-CHOH-CH_2* + H_{Mo}* +O_{Mo}* (3A)	-1.93	0.86
CH_2OH-CHOH-CH_2O* +H_{Mo}* (2A) → CH_2OH-CHO-CH_2O* + 2H_{Mo}* (3B)	-1.57	0.11
CH_2OH-CHO-CH_2O* + 2H_{Mo}* (3B) → CH_2OH-CO-CH_2O* + 3H_{Mo}* (4A)	-0.25	0.79
CH_2OH-CHO-CH_2O* + 2H_{Mo}* (3B) → CH_2OH-CH-CH_2O* + 2H_{Mo}* + O_{Mo}* (4B)	-1.13	0.54
CH_2OH-CHO-CH_2O* + 2H_{Mo}* (3B) → CH_2O-CHO-CH_2O* + 2H_{Mo}* + H_{Cu}* (4C)	-0.44	0.60
CH_2OH-CHO-CH_2O* + 2H_{Mo}* (3B) → CH_2-CHO-CH_2O* + 2H_{Mo}* + OH_{Cu}* (4D)	-0.62	1.48
CH_2OH-CH-CH_2O* +2H_{Mo}* +O_{Mo}* (4B) → CH_2OH-CH=CH_2* +2H_{Mo}* +2O_{Mo}*(5A)	-1.03	0.23
CH_2O-CHO-CH_2O* +2H_{Mo}* +H_{Cu}*(4C) → CH_2O-CH-CH_2O* +2H_{Mo}* +H_{Cu}* +O_{Mo}*(5B)	-0.05	0.64
CH_2OH-CH=CH_2* +2H_{Mo}* +2O_{Mo}*(5A) → (2H_{Mo}*+2O_{Mo}*)(6A) + CH_2OH-CH=CH_2 (g)	1.38	0.00
CH_2OH-CH=CH_2* +2H_{Mo}* +2O_{Mo}*(5A) → CH_2O-CH=CH_2*+2H_{Mo}*+H_{Cu}*+2O_{Mo}*(6B)	-0.38	0.68
CH_2O-CH-CH_2O*+2H_{Mo}*+H_{Cu}*+O_{Mo}*(5B) → CH_2O-CH=CH_2*+2H_{Mo}*+H_{Cu}*+2O_{Mo}*(6B)	-2.05	0.28
CH_2O-CH=CH_2*+2H_{Mo}*+H_{Cu}*+2O_{Mo}*(6B)→CH_2-CH=CH_2*+2H_{Mo}*+H_{Cu}*+2O_{Mo}*+O_{Cu}*(7A)	0.34	1.17
CH_2O-CH=CH_2*+2H_{Mo}*+H_{Cu}*+2O_{Mo}*(6B) → CH_2O-CH-CH_3* + 2H_{Mo}* + 2O_{Mo}*(7B)	0.30	0.63
CH_2O-CH=CH_2*+2H_{Mo}*+H_{Cu}*+2O_{Mo}*(6B) → CHO-CH=CH_2*+2H_{Mo}*+2H_{Cu}*+2O_{Mo}*(7C)	0.03	0.75
(CH_2O-CH-CH_3*+2H_{Mo}*+ 2O_{Mo}*)(7B)+ ½H_2(g)→CH_2O-CH-CH_3*+2H_{Mo}*+2O_{Mo}*+H_{Cu}*(8)	-0.31	0.00
CH_2O-CH-CH_3*+2H_{Mo}*+2O_{Mo}*+H_{Cu}*(8) → CH_2O-CH_2-CH_3* + 2H_{Mo}* + 2O_{Mo}*(9)	-0.33	0.64
CH_2O-CH_2-CH_3* + 2H_{Mo}* + 2O_{Mo}*(9) → CHO-CH_2-CH_3* +H_{Cu}* +2H_{Mo}* +2O_{Mo}*(10A)	0.44	0.54
(CH_2O-CH_2-CH_3*+2H_{Mo}*+ 2O_{Mo}*)(9)+ ½H_2(g)→CH_2O-CH_2-CH_3*+2H_{Mo}*+2O_{Mo}*+H_{Cu}*(10B)	-0.34	0.00
CHO-CH_2-CH_3*+H_{Cu}*+2H_{Mo}*+2O_{Mo}*(10A)→(CHO-CH_2-CH_3*+2H_{Mo}*+2O_{Mo}*)(11A)+½H_2(g)	0.27	0.00
CH_2O-CH_2-CH_3*+2H_{Mo}*+2O_{Mo}*+H_{Cu}*(10B) → CH_2OH-CH_2-CH_3*+2H_{Mo}*+2O_{Mo}*(11B)	0.56	1.34
CHO-CH_2-CH_3*+2H_{Mo}*+2O_{Mo}*(11A) → (2H_{Mo}*+2O_{Mo}*)(6A) + CHO-CH_2-CH_3(g)	0.71	0.00

[a] Each adsorbed reactant or product state in the elementary reactions constitute one discrete state although they are written as having multiple species for clarity

Supplementary Table 6. Zero-point energy corrected reaction energies (ΔE_{ZPE}**)** and forward activation barriers (E_{ZPE}^{act}) for the elementary steps considered in the glycerol decomposition mechanism on Cu sites of the Cu/Mo_2C (0001) catalyst model

**Reaction**[a]	$\boldsymbol{\Delta E_{ZPE}}$ **(eV)**	$\boldsymbol{E_{ZPE}^{act}}$ **(eV)**
CH_2OH-CHOH-CH_2OH (g) + * → CH_2OH-CHOH-CH_2OH* (1)	-0.91	0.00
CH_2OH-CHOH-CH_2OH* (1) → CH_2OH-CHOH-CH_2* + OH* (2A)	-0.65	1.38
CH_2OH-CHOH-CH_2OH* (1) → CH_2OH-CHOH-CH_2O* + H* (2B)	-0.76	0.35
CH_2OH-CHOH-CH_2OH* (1) → CH_2OH-CHO-CH_2OH* + H* (2C)	-0.76	0.27
CH_2OH-CHOH-CH_2OH* (1) → CH_2OH-COH-CH_2OH* + H* (2D)	0.36	0.98
CH_2OH-CHOH-CH_2O* + H* (2B) → CH_2OH-CHOH-CH_2* +H* +O* (3A)	0.18	1.63
CH_2OH-CHOH-CH_2O* + H* (2B) → CH_2OH-CHO-CH_2O* + 2H* (3B)	-0.32	0.53
CH_2OH-CHO-CH_2OH* + H* (2C) → CH_2OH-CHO-CH_2O* + 2H* (3B)	-0.32	0.54
CH_2OH-CHO-CH_2OH* + H* (2C) → CH_2OH-CHO-CH_2* + H* + OH* (3C)	0.09	1.58
CH_2OH-CHO-CH_2OH* + H* (2C) → CH_2OH-CO-CH_2OH* + 2H* (3D)	0.66	0.93
CH_2OH-CHO-CH_2O* + 2H* (3B) → CH_2OH-CHO-CH_2* + 2H* +O* (4A)	0.66	1.79
CH_2OH-CHO-CH_2O* + 2H* (3B) → CH_2OH-CH-CH_2O* + 2H* +O* (4B)	0.70	1.99
CH_2OH-CHO-CH_2O* + 2H* (3B) → CH_2OH-CO-CH_2O* + 3H* (4C)	0.40	0.78
CH_2OH-CHO-CH_2O* + 2H* (3B) → CH_2OH-CHO-CHO* + 3H* (4D)	0.43	1.26
CH_2OH-CHO-CH_2O* + 2H* (3B) → CH_2O-CHO-CH_2O* + 3H* (4E)	0.27	1.13
CH_2OH-CO-CH_2O* + 3H* (4C) → CH_2OH-CO-CH_2* +3H* +O* (5)	-0.71	0.28
CH_2OH-CO-CH_2* +3H* +O* (5) → CH_2OH-CO-CH_3* +2H* +O* (6)	0.42	0.75
CH_2OH-CO-CH_3* +2H* +O* (6) → (2H*+O*) (7) + CH_2OH-CO-CH_3 (g)	0.84	0.00

[a] Each adsorbed reactant or product state in the elementary reactions constitute one discrete state although they are written as having multiple species for clarity

Supplementary Discussion

Correlating Computational Predictions to TPD and HREELS Experimental Results

Gibbs free energy profiles, calculated at a temperature of 350 K and under UHV conditions (P_{gas} = 10^{-9} atm), for the minimum energy pathways of glycerol decomposition on the three active sites investigated are provided in the Supplementary Fig. 13. For the interface sites, only the allyl alcohol formation pathway is shown to improve clarity. On the Mo sites, all O-H and C-O bond cleavages are consistently exergonic, and only the final protonation step is endergonic. This is due to strong adsorption of oxygen atoms on the Mo sites, and these oxygen atoms can only be removed at higher temperatures and by adding excess H_2 as shown in the previous work.[1] Although desorption of propylene is slightly endergonic by 0.06 eV at 350 K, it becomes exergonic above 400 K which agrees well with the TPD experiments that display the largest peak for propylene desorption at 411 K. In our model, propylene is adsorbed on the sites neighboring the adsorbed O and H atoms (see Supplementary Fig. 7, structure 8) for which the adsorption energy is calculated as -1.50 eV (Supplementary Table 3). Our calculations predict that the adsorption of propylene can be further stabilized by 0.6-0.8 eV on sites further away from adsorbed O and H atoms which is consistent with a stronger adsorption energy of -2.95 eV (calculated with RPBE functional) reported for ethylene on Mo_2C (0001) surface[2]. However, such empty Mo sites might not be available in the present case due to the presence of strongly adsorbed O atoms that are dissociation products from glycerol. In addition, the Cu/Mo_2C/Mo(110) surfaces used in the current TPD experiments are pre-dosed with hydrogen which could further affect the availability of empty Mo sites and the stability of the reactant and product on the surface. Free energy calculations under UHV conditions (T= 350 K; P_{H2} = 10^{-9} atm) suggest that the average adsorption free energy of H calculated with reference to the energy of H_2 is -0.49 eV when 8 hydrogen atoms are adsorbed on the Mo sites of the catalyst model used here (which corresponds to a hydrogen coverage on the Mo sites of 0.5 ML). The adsorption of glycerol was further tested in the presence of 8 hydrogen atoms, and we found that the adsorption is destabilized by 0.6 eV in the presence of adsorbed H atoms on neighboring sites (Supplementary Fig. 14). Thus, the free energy of adsorption in the presence of H atoms becomes endergonic (G_{350K}^{ads} = 0.15 eV) compared to an exergonic adsorption of glycerol predicted in the absence of H atoms (Supplementary Fig. 13). This again agrees with the TPD experiments that display a glycerol desorption peak on the hydrogen pre-dosed surfaces at a low temperature of 280 K. However, the presence of H atoms is not expected to change the trend observed in the competing elementary reactions. At an H atom coverage lower than 1 on the Mo sites, the H atoms can easily move between different Mo sites with a small barrier of 0.2-0.3 eV, making the Mo sites available for glycerol dissociation reactions.

All the elementary reactions involved in the allyl alcohol formation pathway at the interface sites are exergonic since these reactions occur on the Mo sites. The role of the Cu atoms is primarily to block the active site for further C-O dissociation. Also, the Cu atoms are active for the isomerization of allyl alcohol to propanal as shown in Fig. 4 of the paper. The free energies of these reactions are slightly above the free energies of the propylene formation pathway, mainly

due to the absence of the third Mo-O bond at the interface. Furthermore, the stability of adsorbed oxygen atoms on similar 3-fold hollow Mo sites was examined at various distances to the Cu/Mo_2C interface. We found that the adsorption of oxygen atoms closer to the interface sites are 0.6-1 eV less stable than those adsorbed farther away from the interface (Supplementary Fig. 15). This suggests that it is easier to remove the adsorbed oxygens at the interface which again agrees with the experimental observations from HREELS spectra that a 0.3 ML Cu/Mo_2C/Mo(110) has a weaker interaction with O than Mo_2C/Mo(110) (Fig. 2 of the paper).

The free energy profile for the formation of acetol on the Cu sites is significantly above the energy profiles for propylene and allyl alcohol formation, and the energy of the most stable state in this pathway (CH_2OH-CO-CH_2*, 5) is only 0.66 eV lower than the initial state (Supplementary Fig. 13). Calculations revealed that the formation of H_2O from the final state (O*+2H*) is exergonic by 0.05 eV at 350 K temperature and thus, the adsorbed oxygen can be easily removed. This is consistent with the results from HREELS spectra that atomic oxygens were not observed on Cu (Fig. 2 of the paper) and also from TPD spectra (Supplementary Fig. 3) that H_2O is easily removed from Cu surfaces. The highest energy state in the free energy profile is found to be the transition state corresponding to the first O-H dissociation process (G_{ZPE}^{act} = 1.00 eV) which appears to be the rate-limiting process for acetol formation.

In order to further examine the removal of adsorbed oxygen atoms from the three different active sites, activation barriers were calculated for the H_2O formation process, both in the presence and absence of excess H_2 (see Supplementary Table 1). On the Mo sites, hydrogenation of either O* or OH* with adsorbed H* was found to be highly endothermic with barriers of about 2 eV (reactions 1 & 2). However, direct dissociation of gas phase H_2 onto O* was found to be favorable with an activation barrier of only 0.38 eV. Similarly, the formation of H_2O via the disproportionation reaction of two neighboring OH* was also found to possess a barrier of only 0.75 eV. These results are consistent with the earlier report by Ren et al. that the adsorbed O on the Mo sites can be removed by excess H_2.[3] On the Cu/Mo_2C interface sites, we examined the possibility of H_2 dissociation on the Cu sites and two subsequent H atom spillovers from the Cu to the interface oxygen adsorbed on the Mo sites to form H_2O. Calculations revealed that the dissociation of gas phase H_2 on Cu is an exothermic process and the spillover of H atoms from Cu to interface O requires overcoming barriers of only about 1 eV, suggesting that these processes are feasible. The direct dissociation of gas phase H_2 onto O* (reaction 9) and OH disproportionation (reaction 10) were also examined at the interface site for which the barriers were calculated as 0.39 and 0.61 eV, respectively. H_2O formation is thus favorable both on the Mo sites and at the interface sites via OH disproportionation provided that empty Mo sites are available at neighboring sites that can promote the formation of OH groups by direct dissociation of gas phase H_2 onto adsorbed O.

On the Cu sites, the activation barriers calculated for H_2O formation in the absence of excess H_2 was found to be >1 eV (reactions 12 & 13 in Supplementary Table 1), however, these barriers are smaller than the corresponding barriers on the Mo sites. The calculated barriers for the stepwise

hydrogenation of O* (1.05 eV) and OH* (1.36 eV) are similar to those reported for different terminations of Cu surfaces[4,5]. Here again, direct dissociation of excess gas phase H_2 onto adsorbed O* (reaction 14) and the OH disproportionation to form H_2O (reaction 15) were found to be more favorable than the stepwise hydrogenation processes. The endothermicity of H_2O desorption from the three sites decreases in the order, Mo site (0.94 eV) > interface site (0.82 eV) > Cu site (0.35 eV), which is consistent with the decrease in the desorption temperature observed in the TPD spectra for H_2O desorption when going from pure Mo_2C to 1 ML Cu/Mo_2C (Supplementary Fig. 3).

These results suggest that H_2O formation from adsorbed O on the three surfaces are possible in the presence of excess H_2 with activation barriers of less than 1 eV. Since the partial pressure of H_2O at UHV conditions is as low as 10^{-12} atm, H_2O can easily desorb from these sites. However, under experimental reactor conditions, i.e., ambient pressure conditions, all the Mo sites could be occupied by oxygen atoms. In fact, constrained ab initio thermodynamic analysis carried out in the presence of an equimolar H_2/H_2O gas phase for our current catalyst model suggested that all the exposed Mo sites are occupied by oxygen atoms at a temperature of 500 K. For this model (1st structure in Supplementary Fig. 16), the oxygen vacancy formation free energy was found to range from 0.4 to 1.2 eV ($O^* + H_2 \rightarrow O_{vac} + H_2O$, T = 500 K; P_{gas}=1 atm) when going from the Cu/Mo_2C interface site to the sites away from Cu. Thus, we examined the possibility of H spillover from Cu to the interface oxygens to form H_2O. The calculated barriers for the 1st and 2nd H spillover process were found to be similar but slightly more favorable on the fully oxygen covered model compared to those calculated for the model with lower oxygen coverage (Supplementary Table 1). The free energy profile calculated at a temperature of 500 K suggested that the removal of interface oxygen is feasible when the P_{H2}/P_{H2O} is slightly above 10^2 which can easily be achieved experimentally. The effective free energy barrier of 1.36 eV corresponds to an approximate oxygen removal rate of 10^{-1} s^{-1}, indicating that the interface oxygens can be removed from an "oxycarbide" surface and the Cu/Mo_2C catalyst should remain active at more practical reactor conditions.

Surface regeneration under UHV condition

To study the role of Cu modifier in catalyst regeneration, sequential TPD experiments were performed on three surfaces, Mo_2C, 0.5 ML Cu/Mo_2C and 1 ML Cu/Mo_2C, between 200 K and 500 K. Supplementary Fig. 5(a) shows the desorption of propylene on the Mo_2C surface. In the second TPD experiment, the propylene desorption peak shifts to 470 K and the intensity reduces, suggesting a lower C-O bond cleavage activity of the Mo_2C surface after the first TPD experiment. In contrast, on the 1 ML Cu/Mo_2C surface (Supplementary Fig. 5(b)), the Cu site maintains activity in three sequential TPD experiments. In Supplementary Fig. 5(c), the 0.5 ML Cu/Mo_2C also loses activity toward allyl alcohol formation after the first TPD experiment. In the second TPD experiment, a small peak of propanal is observed at 402 K (Supplementary Fig. 5(d)). The TPD results under the UHV condition suggest that the Mo_2C surface and Cu-Mo_2C interface are less stable than the Cu surface, which is due to the strong oxophilicity of the Mo site.

However, these experiments were performed under UHV conditions, in which gas-phase H_2 is not involved. In a real catalytic HDO process with a high H_2 pressure, gas-phase H_2 should help remove surface oxygen and maintain a higher hydrogen coverage. In a previous study, Ren et al. studied the HDO reaction of propanal on powder Mo_2C catalyst using a flow reactor.[3] The catalyst quickly deactivated in the absence of H_2. With co-feed H_2, a steady-state propanal conversion of approximately 50% was achieved at 573 K, suggesting that oxygen was removed by H_2. This was also confirmed by the production of water at this temperature. These results are also consistent with DFT results in the current study of lower activation barriers for oxygen removal in the presence of gas-phase H_2 (Supplementary Table 1)."

Supplementary Notes

The quantification results of propylene, acetol, allyl alcohol and propanal were calculated by the following equations:

Propylene yield = $\frac{\theta_{H2}^{sat}}{P_{H2}^{sat}} P_{propylene}^{39} \frac{S_2^{H2}}{S_{39}^{propylene}}$ (Supplementary Equation 1)

Acetol yield = $\frac{\theta_{H2}^{sat}}{P_{H2}^{sat}} P_{acetol}^{43} \frac{S_2^{H2}}{S_{43}^{acetol}}$ (Supplementary Equation 2)

Allyl alcohol yield = $\frac{\theta_{H2}^{sat}}{P_{H2}^{sat}} P_{allyl\ alcohol}^{57} \frac{S_2^{H2}}{S_{57}^{allyl\ alcohol}}$ (Supplementary Equation 3)

Propanal yield = $\frac{\theta_{H2}^{sat}}{P_{H2}^{sat}} P_{propanal}^{58} \frac{S_2^{H2}}{S_{58}^{propanal}}$ (Supplementary Equation 4)

θ_{H2}^{sat} is the saturation coverage of H_2 on the Mo_2C surface and P_{H2}^{sat} is the TPD area of the 2 amu peak from saturation H_2 desorption. S_2^{H2}, $S_{39}^{propylene}$, S_{43}^{acetol}, $S_{58}^{propanal}$ and $S_{57}^{allyl\ alcohol}$ are mass spectrometer sensitivities for H_2, propylene, acetol, propanal and allyl alcohol, respectively. $P_{propylene}^{39}$ and P_{acetol}^{43} were the areas of the 39 amu and 43 amu peaks.

The intensity ratio of the two fragments (m/z 57, m/z 58) from allyl alcohol and propanal was measured by mass spectrometry. Based on the calibration result, the intensity ratio of m/z 58 over m/z 57 is 0.176 for allyl alcohol and 3.07 for propanal. Therefore, $P_{Propanal}^{58}$ and $P_{allyl\ alcohol}^{57}$ were deconvoluted using following equations:

$P_{allyl\ alcohol}^{57} + P_{Propanal}^{58} * \frac{1}{3.07} = P^{57}$ (Supplementary Equation 5)

$P_{Propanal}^{58} + P_{allyl\ alcohol}^{57} * 0.176 = P^{58}$ (Supplementary Equation 6)

P^{57} and P^{58} were the areas of the 57 amu peak and the 58 amu peak from glycerol TPD experiments.

Supplementary References

1. Yu, W., Salciccioli, M., Xiong, K., Barteau, M. a, Vlachos, D. G. & Chen, J. G. Theoretical and Experimental Studies of C–C versus C–O Bond Scission of Ethylene Glycol Reaction Pathways via Metal-Modified Molybdenum Carbides. *ACS Catal.* **4(5),** 1409–1418 (2014).
2. Medford, A. J., Vojvodic, A., Studt, F., Abild-Pedersen, F. & Nørskov, J. K. Elementary steps of syngas reactions on Mo2C(001): Adsorption thermochemistry and bond dissociation. *J. Catal.* **290,** 108–117 (2012).
3. Ren, H., *et al.* Selective hydrodeoxygenation of biomass-derived oxygenates to unsaturated hydrocarbons using molybdenum carbide catalysts. *ChemSusChem* **6,** 798–801 (2013).
4. Gokhale, A. A., Dumesic, J. A. & Mavrikakis, M. On the mechanism of low-temperature water gas shift reaction on copper. *J. Am. Chem. Soc.* **130,** 1402–1414 (2008).
5. Wang, G. C. & Nakamura, J. Structure sensitivity for forward and reverse water-gas shift reactions on copper surfaces: A DFT study. *J. Phys. Chem. Lett.* **1,** 3053–3057 (2010).
